# Inverse Agonistic Action of 3-Iodothyronamine at the Human Trace Amine-Associated Receptor 5

**DOI:** 10.1371/journal.pone.0117774

**Published:** 2015-02-23

**Authors:** Juliane Dinter, Jessica Mühlhaus, Carolin Leonie Wienchol, Chun-Xia Yi, Daniela Nürnberg, Silke Morin, Annette Grüters, Josef Köhrle, Torsten Schöneberg, Matthias Tschöp, Heiko Krude, Gunnar Kleinau, Heike Biebermann

**Affiliations:** 1 Institut für Experimentelle Pädiatrische Endokrinologie, Charité-Universitätsmedizin Berlin, Berlin, Germany; 2 Institute for Diabetes and Obesity, Helmholtz-Zentrum München, German Research Center for Environmental Health, München, Germany; 3 Institut für Experimentelle Endokrinologie, Charité-Universitätsmedizin Berlin, Berlin, Germany; 4 Institut für Biochemie, Molekulare Biochemie, Medizinische Fakultät, University of Leipzig, Leipzig, Germany; Rutgers University, UNITED STATES

## Abstract

**Objective**

Application of 3-iodothyronamine (3-T_1_AM) results in decreased body temperature and body weight in rodents. The trace amine-associated receptor (TAAR) 1, a family A G protein-coupled receptor, is a target of 3-T_1_AM. However, 3-T_1_AM effects still persist in *mTaar1* knockout mice, which suggest so far unknown further receptor targets that are of physiological relevance. TAAR5 is a highly conserved TAAR subtype among mammals and we here tested TAAR5 as a potential 3-T_1_AM target. First, we investigated mouse Taar5 (mTaar5) expression in several brain regions of the mouse in comparison to mTaar1. Secondly, to unravel the full spectrum of signaling capacities, we examined the distinct G_s_-, G_i/o_-, G_12/13_-, G_q/11_- and MAP kinase-mediated signaling pathways of mouse and human TAAR5 under ligand-independent conditions and after application of 3-T_1_AM. We found overlapping localization of mTaar1 and mTaar5 in the amygdala and ventromedial hypothalamus of the mouse brain. Second, the murine and human TAAR5 (hTAAR5) display significant basal activity in the G_q/11_ pathway but show differences in the basal activity in G_s_ and MAP kinase signaling. In contrast to mTaar5, 3-T_1_AM application at hTAAR5 resulted in significant reduction in basal IP_3_ formation and MAP kinase signaling. In conclusion, our data suggest that the human TAAR5 is a target for 3-T_1_AM, exhibiting inhibitory effects on IP_3_ formation and MAP kinase signaling pathways, but does not mediate G_s_ signaling effects as observed for TAAR1. This study also indicates differences between TAAR5 orthologs with respect to their signaling profile. In consequence, 3-T_1_AM-mediated effects may differ between rodents and humans.

## Introduction

The group of trace amine-associated receptors (TAAR) belongs to family A G protein-coupled receptors (GPCRs) [1]. Human and rodent TAARs are expressed in a variety of tissues including several brain regions, kidney, stomach, liver, pancreas, small intestine, pituitary, and leukocytes [2–7]. In addition, it is suggested that TAARs, except for TAAR1, constitute a part of the olfactory system in vertebrates [8–14].

One functional characteristic of TAARs is the high ligand promiscuity, as they can be activated by neurotransmitters [2,15–17], psycho-active drugs [16–19], volatile amines [8,13,20], and trace amines [2,14,19]. In addition, the L-thyroxine-derived thyroid hormone metabolite 3-iodothyronamine (3-T_1_AM), circulating in nano-molar concentrations in human blood [21], has previously been identified as an agonist for rat and mouse Taar1 (mTaar1) [22,23]. 3-T_1_AM was traceable in pico- to nanomolar concentrations in human blood [21,24] and 3-T_1_AM uptake into a high quantity of tissues in rodents at nanomolar concentrations is reliably ascertained, including brain [25–28]. It was shown that 3-T_1_AM reduces body temperature, thus opposing the effects of classical thyroid hormones [22,29]. Further data supported a role of 3-T_1_AM in energy metabolism as its injection into Djungarian hamsters revealed a significant decrease of body mass and changes from carbohydrate to lipid utilization [29]. In a recent study, even a much lower dose of 10 mg/kg 3-T_1_AM (compared to 50 mg/kg), given over a period of 8 days, had a significant influence on weight maintenance in obese mice [30]. Application of 3-T_1_AM in mice resulted in hyperglycemia [31] associated with an increase of plasma glucagon and an endogenous glucose production [32]. Further studies revealed that acute intracerebroventricular (ICV), intra-arcuate nucleus or intraperitoneal administration of 3-T_1_AM induces dose-dependent orexigenic effects on feeding behavior in rodents [33,34]. In addition, ICV administration of 3-T_1_AM improved memory and enhanced curiosity in mice [35] and injection to the pre-optic region of male rats showed a modulation of sleep [36].

However, targeted *mTaar1* gene disruption in mice did not result in significant changes in body weight or temperature regulation [37] and the 3-T_1_AM-induced hypothermic effect is maintained in these mice [38]. Moreover, the pharmacologically inhibitory effects of exogenously administered 3-T_1_AM are rather pointing to a cAMP-repressing effect in contrast to the observed stimulatory effect of 3-T_1_AM on cAMP formation for TAAR1 *in vitro* [22,23].

Such discrepancies indicate that TAAR1 may be not the primary *in vivo* mediator of 3-T_1_AM-induced action.

We here hypothesize that another member of the TAAR group might be a target for 3-T_1_AM and, thereby, be responsible for the observed effects *in vivo*. We focused on TAAR5 as an alternative 3-T_1_AM target, since both TAAR1 and TAAR5 are expressed in primates [39] and TAAR5 is the most highly conserved TAAR subtype among all characterized mammalian species investigated so far. Therefore, expression profiles of mouse Taar1 and Taar5 (mTaar1 and mTaar5) in the brain were investigated with a focus on brain regions that are known to be involved in temperature regulation, like the ventromedial hypothalamus. To unravel the full spectrum of signaling capacities, we examined the distinct G_s_-, G_i/o_-, G_12/13_-, G_q/11_- and MAP kinase-mediated signaling pathways of mouse and human TAAR5 (mTaar5, hTAAR5) under ligand-independent conditions and after application of 3-T_1_AM. To decipher potential molecular reasons of observed differences between signaling of mouse and human TAAR5 we also created and tested chimeric subtype-receptors.

## Materials and Methods

### Expression analysis of mTaar1 and mTaar5 by *in situ* hybridization

Mouse Taar1 and Taar5 were tested for potential overlapping expression in the brain by free floating *in situ* hybridization histochemistry using digoxigenin (DIG)-labeled locked nucleic acid (LNA) probes (Exiqon Inc, Woburn, MA). All studies were approved by and performed according to the guidelines of the Institutional Animal Care and Use Committee (IACUC) of the University of Cincinnati where the respective experiments were performed.

Wild type (WT) C57BL/6 mice were sacrificed by an intra-atrial perfusion with saline, followed by a solution of 4% paraformaldehyde in 0.1 M phosphate buffer (pH 7.4) at 4°C. Brains were then isolated and post-fixed in 4% paraformaldehyde for 16 hours. After equilibration for 48 hours in RNase free 30% sucrose in 0.1 M Tris-buffered saline, tissue was cut into 25 μm sections.

Sections of brain were washed successively with PBS, 0.2 M HCl, and incubated in 0.2% glycin and then 0.1% Triton X-100. Free floating sections were then prehybridized in 1x prehybridzation solution (Sigma Aldrich, St. Louis, MO) and 50% formamide (Sigma Aldrich, St. Louis, MO) for 1 hour at 55°C on a rocking platform. For hybridization, brain sections were incubated for 8 hours with 200 nM concentration of LNA probe in hybridization buffer (Sigma Aldrich, St. Louis, MO) at 57°C. After stringent washing steps with decreasing concentrations of saline-sodium citrate, samples were incubated with 1:500 diluted anti-DIG antibody (goat) at 4°C overnight. In a next step, samples were washed with TRIS-Borate-EDTA-buffer (TBE) and incubated with an avidin-biotin-peroxidase complex (ABC) for 1 hour at room temperature. For visualization of mTaar1, brain sections were stained with 3,3’-diaminobenzidine (DAB) for 5 minutes. Sections were mounted on gelatin-coated glass slides, dried, dehydrated through a graded ethanol series, cleared in xylene and cover-slipped for image collection by light microscopy. mTaar5 samples were stained with anti-DIG antibody as described above, followed by a Dy-Light 488 labeled secondary anti-goat IgG (Jackson ImmunoResearch Laboratories, West Grove, PA). Images were collected by confocal microscopy (Zeiss confocal 710, Carl Zeiss Microscopy GmbH, Jena, Germany).

### Cloning of TAARs and construction of the hTAAR5 chimeras by site directed mutagenesis

All full-length TAAR and control constructs were cloned into the eukaryotic expression vector pcDps and N-terminally tagged with a hemagglutinin (5’ YPYDVPDYA 3’) epitope (HA) for functional assays and determination of cell surface expression, using *Kpn*I and *Spe*I restriction sites. To enhance cell surface expression, hTAAR1 and hTAAR5 were N-terminally fused with the first 21 amino acids of the bovine rhodopsin (Rho-tag) as previously described [8,20,40].

hTAAR5 chimeras were generated by exchanging 8 amino acids differing between human and mouse receptors using site-directed mutagenesis. For each step, a PCR was performed using overlapping oligonucleotides containing the respective amino acid exchange. Mutagenesis was performed based on the above described full-length hTAAR5 sequence, cloned into the eukaryotic expression vector pcDps and N-terminally tagged with a hemagglutinin epitope and Rho-tag. All plasmids were sequenced and verified with BigDye-terminator sequencing (PerkinElmer Inc., Waltham, MA) using an automatic sequencer (ABI 3710 XL; Applied Biosystems, Foster City, CA).

### Cell culture and transient transfection

For determination of signal transduction properties (cAMP, inositol trisphosphate (IP)-3-, RhoA- and MAP kinase reporter gene assays), HEK293 cells (human embryonic kidney cells) [41] (American Type Culture Collection, LGC Standards GmbH, Wesel, Germany) were cultured in Minimum Essential Medium Earle´s (MEM) (Biochrom AG, Berlin, Germany) supplemented with 10% FBS (PAA Laboratories GmbH, Pasching, Austria), 100 U/mL penicillin, 100 μg/mL streptomycin (Biochrom AG, Berlin, Germany) and 2 mM L-glutamine (Invitrogen, Carlsbad, CA) at 37°C with 5% CO_2_.

Cell surface expression studies were conducted in COS-7 cells (African Green Monkey SV40-transfected kidney fibroblast cell line) [42] (American Type Culture Collection, LGC Standards GmbH, Wesel, Germany), which are more robust facing the numerous washing steps. COS-7 cells were cultured in Dulbecco´s Modified Eagle Medium (DMEM) (Biochrom AG, Berlin, Germany) supplemented identically as MEM. Transient transfection was performed in the respective supplement-free medium and media were changed 16 hours after transfection. For obtaining mock data, cells were transiently transfected with the empty vector pcDps.

For cAMP accumulation assays, HEK293 cells were cultured in 48 well plates (3.75 x 10^4^ cells/well), and transiently transfected with 83 ng DNA using 0.9 μL Metafectene (Biontex, Munich, Germany) per well, 24 hours after seeding.

For reporter gene assays (G_12/13_, MAP kinase and IP_3_), HEK293 cells were seeded in 96-well plates (1.5 x 10^4^ cells/well) or 48-well plates (3.75 x 10^4^ cells/well, for pertussis toxin (PTX) sensitivity experiments). 28 hours post-seeding, receptor DNA was co-transfected with the respective reporter construct (Promega, Fitchburg, WI), using 0.9 μL Metafectene and a total amount of 167 ng DNA in equal ratios per well.

For internalization studies, COS-7 cells were seeded in 48 well plates (3.75 x 10^4^ cells/well) and transiently transfected with 167 ng DNA using 1 μL Metafectene per well.

Ligands were purchased from Sigma Aldrich (St. Louis, MO). 3-T_1_AM was purchased from Santa Cruz Biotechnology (Dallas, Texas).

### Determination of cell surface expression

To determine ligand-induced internalization and cell surface expression of the chimeric hTAAR5, a cell surface ELISA (enzyme-linked immunosorbent assay) was performed. Therefore, cells were transiently transfected with N-terminally HA-tagged receptors. For internalization studies, cells were additionally stimulated for six hours with 10 μM 3-T_1_AM [22], 72 hours post-transfection. For samples at time point zero, incubation media were immediately replaced and cells fixed with 4% paraformaldehyde and blocked with 10% FBS overnight.

Cells were probed with a biotin labeled Anti-HA antibody (Roche, Basel, Switzerland) (1:200) and detected with horseradish peroxidase-labeled Streptavidin (BioLegend, London, UK) (1:2,500). Color reaction was achieved by adding the substrate o-phenylendiamine (Sigma Aldrich, St. Louis, MO) solved in a buffer composed of 0.1 M citric acid and 0.1 M sodium hydrogen-phosphate enriched with hydrogen peroxide. Reaction was stopped with sodium sulfite-oversaturated 1 M hydrogen chloride. Absorption was measured at 492 / 620 nm by using Anthos Reader 2001 (Anthos Labtech Instruments, Salzburg, Austria).

### Determination of G_s_ and G_i/o_ activation by cAMP accumulation assays

G_s_ and G_i/o_ signaling were measured by a competitive cAMP accumulation assay based on the AlphaScreen technology (Perkin-Elmer Life Science, Boston, MA). Stimulation was performed in stimulation buffer composed of MEM and 1 mM 3-isobutyl-1-methylxanthine (IBMX, Sigma Aldrich, St. Louis, MO) as phosphodiesterase inhibitor, 40 hours post-transfection.

For measurement of G_s_ activation, HEK293 cells were incubated for 45 minutes with 10 μM 3-T_1_AM and/or 100 μM dimethylethylamine (DMEA, Sigma Aldrich, St. Louis, MO). mTaar1 stimulated with 3-T_1_AM and mTaar5 stimulated with DMEA served as positive assay controls with less elaborate handling compared to other known mTaar5 agonists [20,22]. For G_i/o_ signaling analysis, cells were co-stimulated with 50 μM forskolin (AppliChem GmbH, Darmstadt, Germany) to stimulate the overall adenylyl cyclase. Basal G_i/o_ activity of TAAR was determined in comparison to FSK-stimulated mock transfection.

Stimulation was performed in triplicates at 37°C with 5% CO_2_ saturated air and was stopped by aspirating the medium. Cells were then lysed for 1.5 hours at 4°C on a shaking platform with cell lysis buffer containing 5 mM HEPES, 0.1% BSA, 0.3% Tween 20, and 1 mM IBMX (pH 7.4). Intracellular cAMP accumulation was determined according to the manufacturers‘ protocol and as previously described [43].

### Determination of G_12/13_, G_q/11_ and MAP kinase activation via luciferase reporter gene assay

G_12/13,_ MAP kinase (MAPK) and G_q11_/phospholipase C (G_q/11_) signaling were measured via a luciferase reporter gene assay (G_12/13_: RhoA-luc; MAPK: SRE-luc; G_q/11_: IP3-luc) (Promega, Fitchburg, WI). For measurement of G_12/13_ signaling *(RhoA-luc)*, cells were co-transfected with a reporter construct containing the firefly luciferase gene under the control of a serum response factor response element (SRF-RE) (pGL4.34) and either the receptor construct or the empty vector plasmid DNA. Transcription of luciferase reporter gene is activated in response to RhoA GTPase activation. For indirect measurement of the MAP kinase pathways (SRE-luc), cells were co-transfected with a reporter construct containing the firefly luciferase reporter gene and a serum response element (SRE) (pGL4.33), and either receptor or empty vector plasmid DNA. SRE drives the transcription of luciferase reporter gene in response to activation of MAP kinase signaling pathway. For measurement of G_q/11_ signaling pathway (IP3-luc), cells were co-transfected with the nuclear factor of activated T-cells (NFAT) DNA (pGL4.30), a reporter construct containing the firefly luciferase gene under the control of the NFAT response element (NFAT-RE), and either receptor or empty vector plasmid DNA. Transcription of luciferase is driven by NFAT-RE in response to IP3 formation.

To discriminate between PTX-sensitive and PTX-insensitive basal signaling, transiently transfected cells were incubated with 500 nM PTX, 20 hours prior to ligand stimulation. Supplement-free MEM was added to the untreated cells.

Two days post-transfection, cells were washed with PBS (Biochrom AG, Berlin, Germany) and stimulated for 6 hours with 10 μM 3-T_1_AM and/or 100 μM DMEA in supplement-free MEM at 37°C with 5% CO_2_ air. Reactions were terminated by aspirating the medium.

Cells were lysed for 15 minutes on a shaking platform at room temperature, using 1x passive lysis buffer (Promega, Fitchburg, WI). Measurement was conducted with automatic luciferase substrate injection of 40 μL in a black 96 well plate using a Berthold Microplate Reader (Berthold Technologies GmbH & Co KG, Bad Wildbad, Germany).

### Data analysis

Basal state is shown as fold over basal mock transfection, and stimulation is referred to fold over ligand-stimulated mock with the respective substance to omit endogenous effects. Bar graphs and concentration-response curve with mean ± SEM as well as statistical analyses were generated using GraphPad Prism Version 6.0 (GraphPad Software, San Diego, CA).

## Results

### Overlapping tissue expression of mTaar5 and mTaar1 in mouse brain

**Fig 1:**
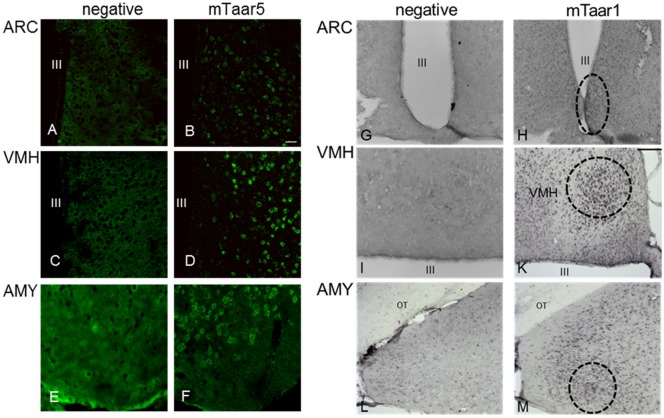
Expression of mTaar1 and mTaar5 in various murine brain regions. Transcript expression studies were analyzed by *in situ* hybridization using a LNA (locked nucleic acid) probe. C57BL/6 mouse brains were sectioned and treated with the corresponding LNA probes. Signals were visualized by an avidin-biotin complex using DY-light 488 streptavidin **(A-F)** or DAB (3,3’-diaminobenzidin) staining **(G-M)**. III = third ventricle; OT = optical tract; DY-light 488 streptavidin labeled samples are shown with a 40-fold, DAB stained sections are depicted with a 20-fold magnification. Bar scale in (B) equals 100 μm, bar scale in (K) equals 200 μm. **(A-F)**: mTaar5 expression; **(A), (C)** and **(E)** represent negative controls using a scrambled LNA probe showing homogenous staining. **(B), (D)** and **(F)** show expression of mTaar5 in arcuate nucleus (ARC), in ventromedial hypothalamus (VMH) and amygdala, respectively. **(G-M)**: mTaar1 expression, the brain regions of interest are highlighted by circles. **(G)**, **(I)** and (**L)** represent negative controls using a scrambled LNA probe. **(K)** and **(M)** show expression of mTaar1 in mice brains in VMH and amygdala, respectively. **(H)** No expression could be detected in the ARC.

mTaar5 expression has been demonstrated in the olfactory epithelium [8,11,44]. Because it is well known that distinct areas in the hypothalamus are important for temperature and body weight regulation [45–47] we analyzed the expression of mTaar1 and mTaar5 in distinct hypothalamic regions. We performed *in situ* hybridization with double DIG-labeled LNA probes on mouse brain sections to achieve high specificity and sensitivity with minimal risk for genomic DNA contamination. A scrambled nonsense LNA probe served as negative control for the respective tissue sections showing no specific signals (Fig. 1 A, C, E, G, I, L).

Signals specific for mTaar5 were detected in the arcuate nucleus (ARC) (Fig. 1B), the ventromedial hypothalamus (VMH) (Fig. 1D), and the amygdala (Fig. 1F). No mTaar5 expression was observed in any other hypothalamic region. Expression of mTaar1 was detected in the VMH (Fig. 1K) and the amygdala (Fig. 1M) of mouse brain but not in the ARC (Fig. 1H). Our data indicate overlapping expression of mTaar5 and mTaar1 in the VMH and the amygdala.

### Mouse Taar5 but not hTAAR5 shows basal G_s_-mediated signaling

**Fig 2:**
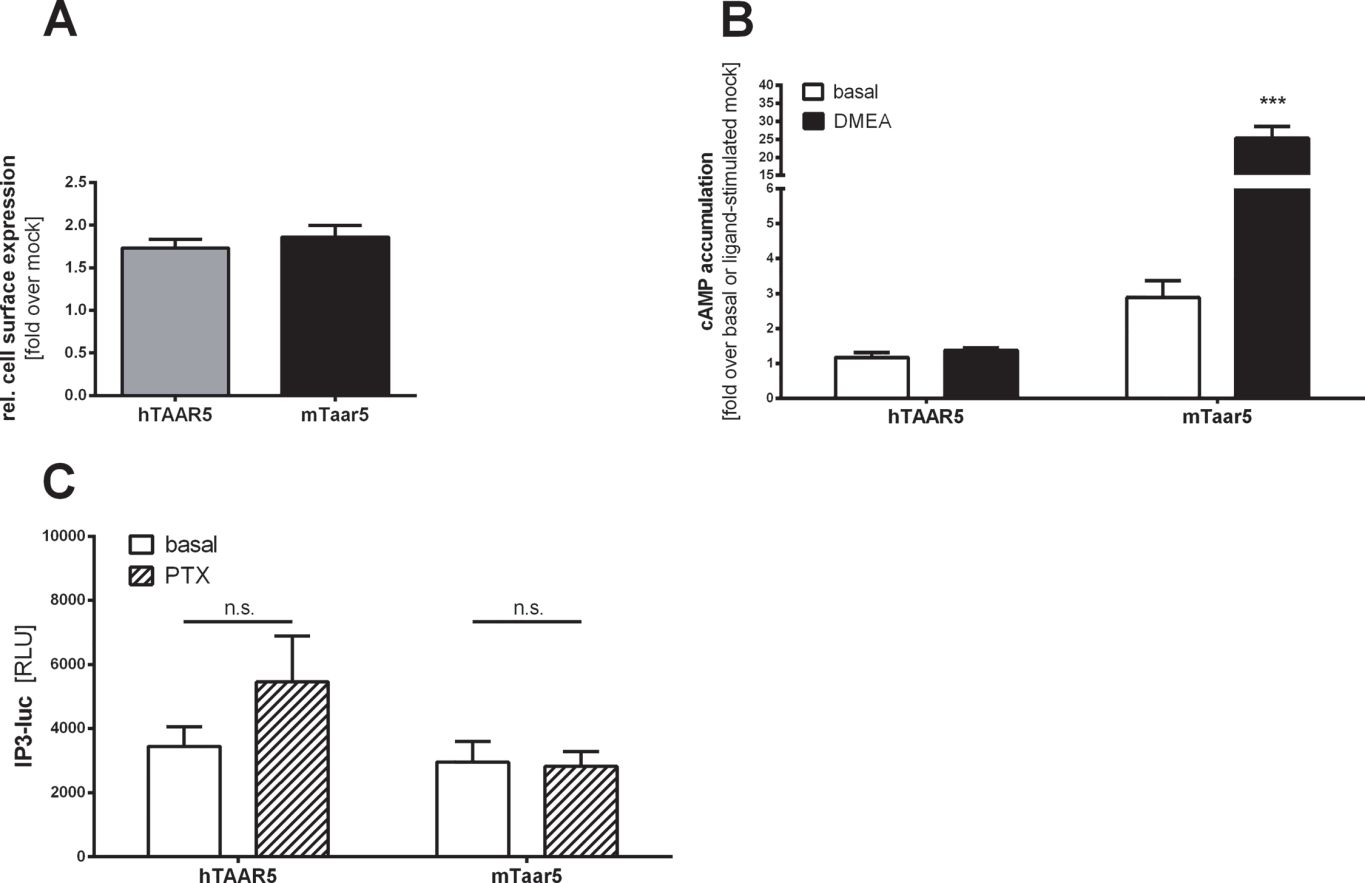
Functional characterization of mouse and human TAAR5 in cAMP and IP_3_ assays. **(A)** Cell surface expression was tested using an ELISA. Mouse and human TAAR5 were N-terminally HA-tagged and transiently transfected with COS-7 cells. Results are depicted as mean ± SEM obtained from 3 independent assays measured in 4 replicates. Data are presented as fold over mock transfection. An unpaired two-tailed t-test was performed which showed no significant difference of cell surface expression between both receptor orthologs. **(B)** HEK293 cells expressing mouse or human TAAR5 were stimulated with 100 μM DMEA. The cAMP accumulation was measured by competitive cAMP assay based on AlphaScreen technology. Results are depicted as either fold over basal mock or fold over DMEA stimulated mock transfection. Data are shown as mean ± SEM from n ≥ 3 independent experiments with 3 or more replicates. Statistical analyses were carried out with an unpaired two-tailed Welch-corrected t-test; ***p ≤ 0.001, compared to the respective basal activity. **(C)** HEK293 cells transiently expressing hTAAR5 or mTaar5 were incubated with or without 500 nM pertussis toxin (PTX), 20 hours prior to measurement. Supplement-free MEM was added to the untreated cell. IP3-luc levels were determined without (basal) and with PTX treatment. Results are presented as relative light units (RLU). Data were obtained from 3 to 6 independent experiments measured in triplicates and are shown as mean ± SEM. An unpaired two-tailed Welsh-corrected t-test was used for statistical analyses; *p ≤ 0.05; **p ≤ 0.01; ***p ≤ 0.001. Human TAAR5 shows elevated basal IP3-luc activity, which was maintained after treatment with PTX pointing to a basal G_q/11_ activity. Basal IP3-luc activity of mTaar5 was not significantly influenced by PTX treatment.

To analyze signal transduction properties of mTaar5 and hTAAR5, both orthologs were expressed in HEK293 cells and showed comparable cell surface expression levels in ELISA (Fig. 2A).

As already reported [8,20,48], mTaar5 displayed basal activity in the G_s_/adenylyl cyclase pathway (G_s_) (Table 1, Fig. 2B). In contrast, hTAAR5 revealed no significant basal activity in the cAMP assay (Fig. 2B). DMEA (100 μM) induced a robust cAMP signal via mTaar5 but not via hTAAR5 (Table 1, Fig. 2B). This indicates significant species-specific differences in TAAR5 signaling.

**Table 1:** Signaling pathways, basal activity and stimulation with 3-T_1_AM at mouse and human TAAR5.

TAAR subtype	second messenger signaling	basal signaling *[fold over basal mock]*	**ligand**	ligand stimulated signaling *[fold over stimulated mock]*
hTAAR1	cAMP level	13.3 ± 5.1	**3-T_1_AM (10 μM)**	204.4 ± 25.2 **
hTAAR5	cAMP level	1.9 ± 0.9	**3-T_1_AM (10 μM)**	0.7 ± 0.2
	IP3-luc	3.6 ± 0.2	**3-T_1_AM (10 μM)**	2.7 ± 0.2 **
	cAMP inhibition	1.0± 0.1	**3-T_1_AM (10 μM)**	1.0 ± 0.2
	SRE-luc	6.2 ± 0.8	**3-T_1_AM (10 μM)**	4.0 ± 0.6 *
	RhoA-luc	1.6 ± 0.2	**3-T_1_AM (10 μM)**	1.4 ± 0.1
mTaar5	cAMP level	2.2 ± 0.8	**3-T_1_AM (10 μM)**	3.5 ± 1.8
	IP3-luc	3.5 ± 0.3	**3-T_1_AM (10 μM)**	3.5 ± 0.4
	cAMP inhibition	1.9 ± 0.2	**3-T_1_AM (10 μM)**	1.7 ± 0.1
	SRE-luc	3.1 ± 0.4	**3-T_1_AM (10 μM)**	2.5 ± 0.5
	RhoA-luc	1.6 ± 0.1	**3-T_1_AM (10 μM)**	1.3 ± 0.1
hTAAR5	cAMP level	1.2 ± 0.01	**DMEA (100 μM)**	1.4 ± 0.1
mTaar5	cAMP level	2.9 ± 0.5	**DMEA (100 μM)**	25.4 ± 3.2 ***

HEK293 cells expressing hTAAR5 or mTaar5 were stimulated with either 10 μM 3-T1AM or 100 μM DMEA. Values indicate cAMP accumulation or luciferase activity (IP3-luc, RhoA-luc, SRE-luc), and are depicted as mean ± SEM fold over basal or ligand-stimulated mock. Treatment with DMEA, serving as assay control in Gs signaling, showed a stimulation of about 8-fold over basal for mTaar5 but not for hTAAR5. For Gq/11 signaling, the basal activity of hTAAR5 and mTaar5 are statistically significant (***p ≤ 0.001) compared to mock transfection (original data of hTAAR5: 19489 ± 2925 RLU; mTaar5: 33396 ± 1434; vs. mock transfection: 5809 ± 859 RLU, respectively). For Gs signaling, the increased basal value of hTAAR1 is statistically significant (***p ≤ 0.001) but not for hTAAR5 compared to mock transfection (original data of hTAAR1: 3 ± 0.6 nM cAMP; hTAAR5 0.6 ± 0.2 nM cAMP; vs. mock transfection: 0.3 ± 0.08 nM cAMP, respectively). Furthermore, mTaar5 showed elevated, but not significant basal values in the Gs signaling pathway compared to mock transfection (p = 0.1 with raw values of mock 1.2 ± 0.4 nM cAMP and mTaar5 3.1 ± 1.0 nM cAMP). Both TAAR5 orthologs showed elevated basal activity in the SRE reporter assay (MAPK signaling). Results of statistical analyses with stimulated value compared to basal value of the respective TAAR are shown analogously to the figures. Unpaired two-tailed Welsh-corrected t-tests were performed

*p ≤ 0.05

**p ≤ 0.01

***p ≤ 0.001.

HEK293 cells expressing hTAAR5 or mTaar5 were stimulated with either 10 μM 3-T_1_AM or 100 μM DMEA. Values indicate cAMP accumulation or luciferase activity (IP3-luc, RhoA-luc, SRE-luc), and are depicted as mean ± SEM fold over basal or ligand-stimulated mock. Treatment with DMEA, serving as assay control in G_s_ signaling, showed a stimulation of about 8-fold over basal for mTaar5 but not for hTAAR5. For G_q/11_ signaling, the basal activity of hTAAR5 and mTaar5 are statistically significant (***p ≤ 0.001) compared to mock transfection (original data of hTAAR5: 19489 ± 2925 RLU; mTaar5: 33396 ± 1434; vs. mock transfection: 5809 ± 859 RLU, respectively). For G_s_ signaling, the increased basal value of hTAAR1 is statistically significant (***p ≤ 0.001) but not for hTAAR5 compared to mock transfection (original data of hTAAR1: 3 ± 0.6 nM cAMP; hTAAR5 0.6 ± 0.2 nM cAMP; vs. mock transfection: 0.3 ± 0.08 nM cAMP, respectively). Furthermore, mTaar5 showed elevated, but not significant basal values in the G_s_ signaling pathway compared to mock transfection (p = 0.1 with raw values of mock 1.2 ± 0.4 nM cAMP and mTaar5 3.1 ± 1.0 nM cAMP). Both TAAR5 orthologs showed elevated basal activity in the SRE reporter assay (MAPK signaling). Results of statistical analyses with stimulated value compared to basal value of the respective TAAR are shown analogously to the figures. Unpaired two-tailed Welsh-corrected t-tests were performed; *p ≤ 0.05; **p ≤ 0.01; ***p ≤ 0.001.

**Fig 3:**
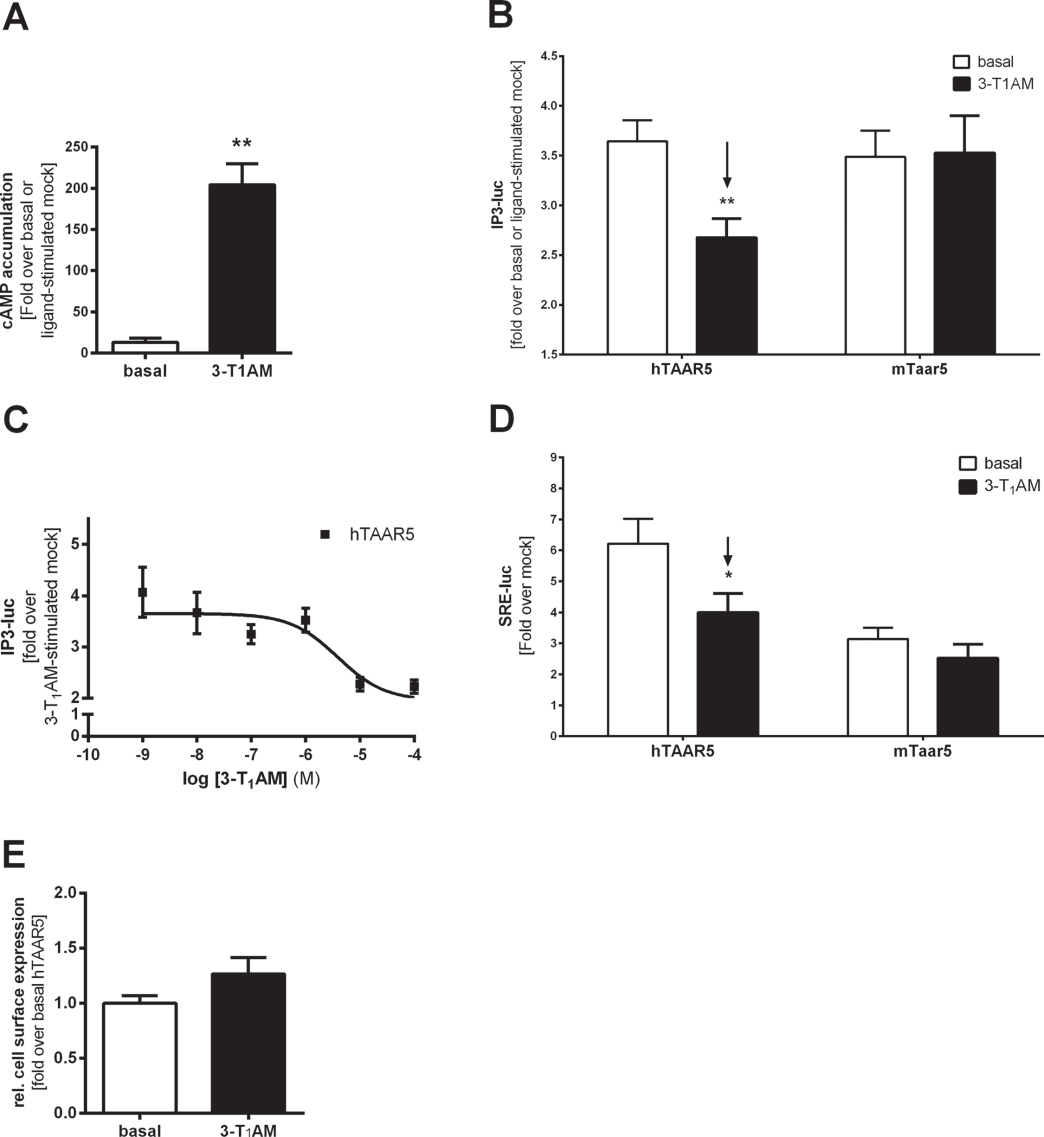
Signaling parameters of human and murine TAAR5 after treatment with 3-T_1_AM. **(A)** HEK293 cells expressing human TAAR1 were stimulated with 10 μM 3-T_1_AM. For G_s_ signal determination cAMP accumulation was measured. Results are depicted as fold over basal mock or fold over 3-T_1_AM-stimulated mock. Data is shown as mean ± SEM from n ≥ 3 independent experiments with 3 or more replicates. 3-T_1_AM is a potent agonist for hTAAR1 (**p < 0.01). Statistical analysis was carried out with an unpaired two-tailed Welch-corrected t-test. **(B)** HEK293 cells transiently expressing hTAAR5 or mTaar5 were stimulated with 10 μM 3-T_1_AM and IP3-luc levels were determined. Results are presented as either fold over basal mock transfection for basal value or fold over 3-T_1_AM stimulated mock. An unpaired two-tailed Welsh-corrected t-test was used for statistical analyses, **p ≤ 0.01. Data are obtained from 3 to 6 independent experiments measured in at least triplicates and are shown as mean ± SEM. **(C)** Human TAAR5 was stimulated with 3-T_1_AM concentrations ranging from 1 nM to 100 μM. The concentration-dependent IP3-luc signaling curve indicated the inverse agonism of 3-T_1_AM at hTAAR5 with an EC_50_ value of 4.4 ± 0.9 μM. **(D)** MAP kinase activation was determined by luciferase activity in a luciferase reporter gene assay (SRE-luc). HEK293 cells were co-transfected with a reporter construct containing a serum response element linked to the firefly luciferase reporter gene and in combination with the different receptor constructs, respectively. Cells were stimulated with 10 μM 3-T_1_AM and SRE-luc levels were determined. Results are presented as mean ± SEM as either fold over basal mock transfection for basal value or fold over 3-T_1_AM-stimulated mock. An unpaired two-tailed Welsh-corrected t-test was performed for statistical analyses; *p ≤ 0.05. **(E)** Cell surface expression studies of hTAAR5 were conducted in COS-7 cells for 6 hours after stimulation with or without 10 μM 3-T_1_AM using an ELISA. Results are depicted as mean ± SEM obtained from 3 independent assays measured in 4 replicates. Data are presented as fold over basal hTAAR5. An unpaired two-tailed t-test with Welch-correction was performed.

Next, we unraveled the signal transduction pathway for hTAAR5 and tested G_q/11_ via a reporter gene assay (IP3-luc). Interestingly, we found high basal activity for both hTAAR5 and mTaar5 (Figs. 2C, 3B, Table 1). This basal activity was pertussis toxin (PTX)-insensitive (Fig. 2C). Furthermore, neither basal activity of the G_i/o_ adenylyl cyclase (G_i/o_) nor of the G_12/13_ signaling was detected (Table 1).

### 3-T_1_AM decreases basal IP3 level of hTAAR5

3-T_1_AM acts as an agonist at rat Taar1 [22] and also hTAAR1 (Fig. 3A). In contrast, concentrations ranging from 10^–5^ M to 10^–7^ M 3-T_1_AM did not induce cAMP accumulation in HEK293 cells transfected with hTAAR5 or mTaar5 (Table 1).

Next, we tested whether 3-T_1_AM exerts effects on IP_3_ signaling. Unexpectedly, 3-T_1_AM significantly reduced basal IP_3_ formation to ~70% of the signal obtained for non-treated cells (Fig. 3B). This decrease of basal IP_3_ formation was concentration-dependent with an EC_50_ value of 4.4 ± 0.9 μM (Fig. 3C).

Using an indirect measure of the MAP kinase pathway activation, we co-transfected hTAAR5 or mTaar5 with an SRE reporter construct. Both TAAR5 orthologs showed increased basal activity in the SRE reporter assay (Fig. 3D, Table 1). Similar to the data observed for IP_3_ formation, 3-T_1_AM application resulted in a robust reduction of signaling in hTAAR5 but not in mTaar5 transfected cells (Fig. 3D). These findings point to an inverse agonistic effect of 3-T_1_AM at hTAAR5. Moreover, DMEA showed an inverse agonistic effect in the G_q/11_ pathway on mTaar5, but not on hTAAR5 (S1 Fig.). Co-treatment of DMEA and 3-T_1_AM at hTAAR5 revealed no effect of DMEA on the 3-T_1_AM-induced basal signalling inhibition. On the other hand, 3-T_1_AM did not modulate the inverse agonistic action of DME at mTaar5 (S1 Fig.).

To ensure that the signal reduction after 3-T_1_AM incubation is not due to internalization of the receptor, we investigated cell surface expression following 3-T_1_AM incubation for 6 hours. As shown in Fig. 3E, 3-T_1_AM treatment had no significant influence on hTAAR5 cell surface expression.

In summary, our data indicate profound differences between mTaar5 and hTAAR5, specifically in basal signaling properties, DMEA-induced cAMP accumulation, and 3-T_1_AM-modified basal activities.

### Substitutions of mTaar5-specific amino acids in hTAAR5

**Fig 4:**
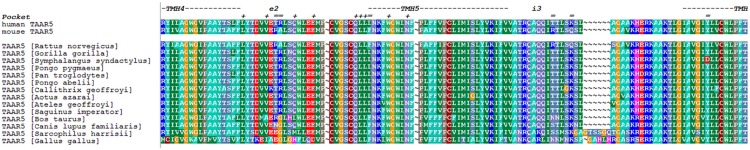
Partial sequence alignment between TAAR5 subspecies. This alignment compares amino acids of different TAAR subspecies in a region between transmembrane helix (TMH) 4 and TMH6. Particular background colors indicating biophysical properties of the amino acid side chains: black—proline, blue—positively charged, cyan/green—aromatic and hydrophobic, green—hydrophobic, red—negatively charged, gray—hydrophilic, dark-red—cysteines, magenta—histidine. The putative helix dimensions and loop regions are assigned according to observable properties in the crystal structure of the inactivated β_2_-adrenergic receptor conformation (pdb entry code 2RH1). Furthermore, in homology to the ligand binding regions of β-adrenergic receptors, amino acid positions covering the putative ligand binding region of TAARs are marked with a plus (+) (see also [43]). Divergent residues between mouse and human TAAR5 participating either in forming the potential extracellular ligand binding site or the intracellular effector binding region are indicated by ‘ = ’. These residues were suggest in this study for substitutions between mouse and human TAAR5 and were also visualized on the structural model in Fig. 5. The overall identity between human and mouse amino acid sequence is 87% (Blossum62 identity matrix). For alignment visualization the program *BioEdit* was used.

**Fig 5:**
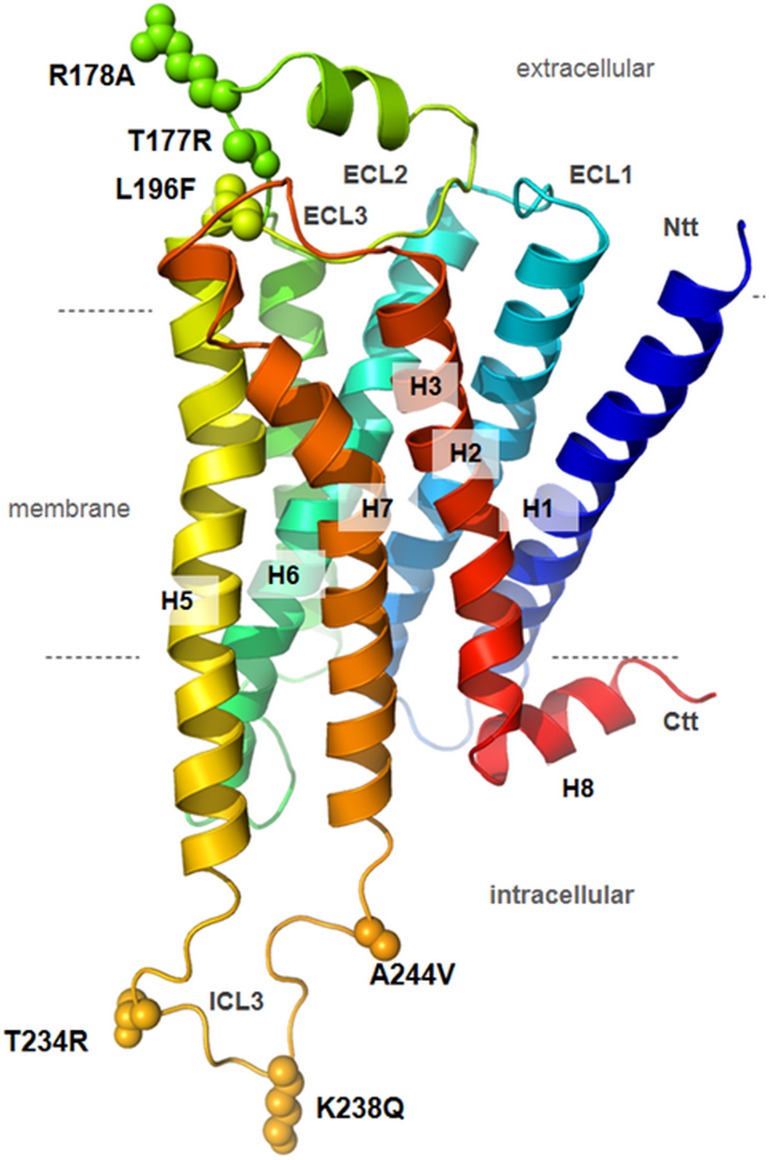
Structural homology model of hTAAR5 with highlighted amino acid positions that were substituted to design chimeric human-murine receptors. The design of a structural homology model of hTAAR5 was already reported [43]. We used this 3D information to visualize the amino acids differing between human and mice TAAR5 in accordance to observed differences in the sequence of both species (Fig. 4) with a focus on residues that are in spatial proximity to the extracellular ligand binding part or the intracellular effector binding region. The human wild type amino acids (shown by atom spheres) and the equivalent residues in mouse are provided as mutations in the labels. H1–7 = seven-transmembrane helices 1–7; H8 = eighth intracellular helix; ECL = extracellular loop, ICL = intracellular loop, Ntt = N-terminal tail; Ctt = C-terminal tail.

To unravel the molecular details of these species-specific differences of TAAR function, we generated chimeric receptors based on differences in the amino acid sequence of both receptors. For this purpose an amino acid sequence alignment was used to predict residues that are potentially involved in ligand binding and signaling (for detailed substitutions see Table 2, and Figs. 4, 5). These specific residues were substituted in three multiple combinations from mTaar5 into corresponding positions of hTAAR5. In total, six single mutations were substituted at corresponding positions, three located in the extracellular loop ECL2 and three in the intracellular loop ICL3 (Table 2 and Fig. 5).

**Table 2:** Overview of the amino acid substitutions between human and mice for construction of chimeric TAAR5.

Construct	Substitution					
	extracellular			intracellular		
chim1	T177R	R178A	L196F			
chim2				T234R	K238Q	H246R
chim3	T177R	R178A	L196F	T234R	K238Q	H246R

Based on an amino acid sequence alignment between mouse and human TAAR5, the six residues depicted here (see also Fig. 5) from mTaar5 were identified to be species-specific and potentially involved in binding and signaling capacities. They were finally substituted in three multiple combinations from mTaar5 into corresponding positions of hTAAR5, subdivided into mutations at the extra- and intracellular site. Combinations of multiple-mutations were designed to unravel potential effects that are dependent on simultaneous modifications at both receptor sites.

**Fig 6:**
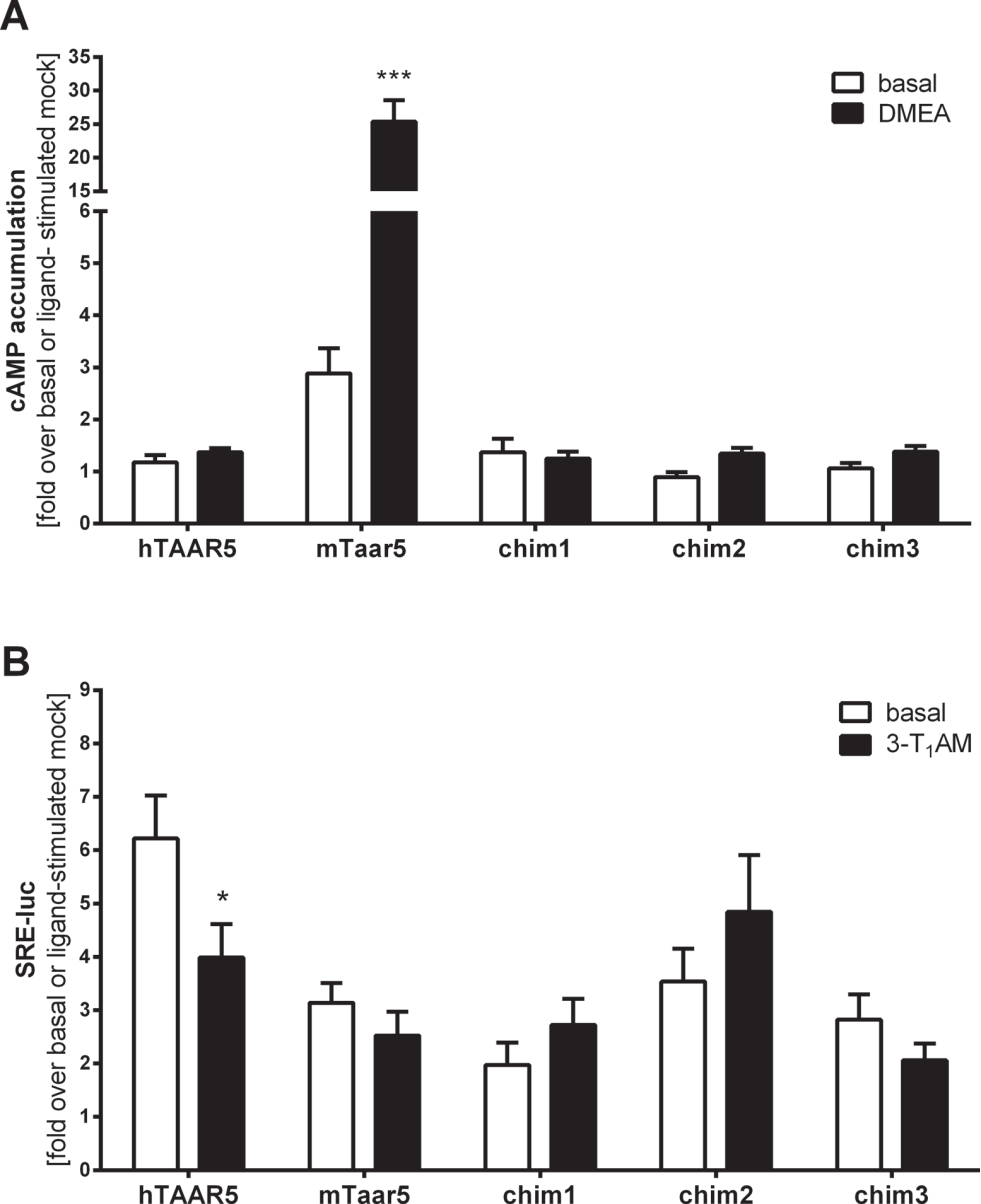
MAP kinase activation and G_s_ signaling parameters of wild type TAAR5 and chimeric receptors. **(A)** HEK293 cells expressing mouse or human TAAR5 or chimeric receptors (see Table 2 for details) were stimulated with 100 μM DMEA. The cAMP accumulation was measured by competitive cAMP assay based on AlphaScreen technology. Results are depicted as either fold over basal mock or fold over DMEA stimulated mock transfection. Data are shown as mean ± SEM from n ≥ 3 independent experiments with three or more replicates. Statistical analyses were carried out with an unpaired two-tailed Welch-corrected t-test; ***p ≤ 0.001, compared to the respective basal activity. **(B)** MAP kinase activation was reported by luciferase activity in a luciferase reporter gene assay (SRE-luc). HEK293 cells were co-transfected with a reporter construct containing a serum response element and the firefly luciferase reporter gene, and the different receptor constructs. Cells were stimulated with 10 μM 3-T_1_AM and SRE-luc levels were determined. Results are presented as mean ± SEM as either fold over basal mock transfection for basal value or fold over 3-T_1_AM-stimulated mock. An unpaired two-tailed Welsh-corrected t-test was performed for statistical analyses; *p ≤ 0.05.

Our experiments demonstrate that none of the combined substitutions transfers the known cAMP signaling properties from murine to human TAAR5 (Fig. 6A), with respect to the high basal activity or the capacity to induce cAMP accumulation by DMEA. The increased basal cAMP accumulation and ligand induced stimulation by DMEA at mTaar5 was not observed for any chimeric construct, including a full six amino-acid substitution that combines both all extra- and intracellular substitutions (Table 2, chim3). Furthermore, the constructs were tested in the MAPK signaling pathways due to higher signal ratios compared to the background. In this SRE-reporter assay, each modification at hTAAR5 including the minimal multiple-mutants with three side chain substitutions led to a decreased basal SRE activity (Fig. 6B). The decrease of SRE-luc signaling after application of 3-T_1_AM as observed for hTAAR5 was consequently also abolished at the chimeric receptors with the lowered basal activity.

## Discussion

The main objective of this study was the characterization of TAAR5 as a potential receptor target for 3-T_1_AM that might account for 3-T_1_AM-mediated physiological effects. In this study, we tested whether TAAR5 stimulation by the putative ligand 3-T_1_AM and the known agonist DMEA activate distinct signaling pathways via G_s_, G_i/o_, G_12/13_ and G_q/11_ coupling as well as MAP kinase pathway activation.

### Co-expression of mTaar1 and mTaar5 in brain regions

First of all, we demonstrated that mTaar5 is expressed in specific regions in the brain that are responsible for weight regulation as well as for the control of body temperature [45–47] like the VMH. As several studies indicate that AMP-activated protein kinase (AMPK) in the VMH plays a role in thermogenesis of brown adipose tissue, we speculated that activation of mTaar5 might, therefore, explain the observed effect on body temperature after 3-T_1_AM application in wild type and mTaar1 deficient mice [45,46,49].

It is commonly accepted that TAARs represent a second class of olfactory receptors in the olfactory epithelium of vertebrates [8–14]. Except for TAAR1, all TAAR subtypes are expressed in the olfactory epithelium with TAAR5 exhibiting the highest expression levels. TAARs are hypothesized to be pheromone receptors even in humans, but the mTaar5 is still more sensitive to volatile amines than its human orthologe [44].

### 3-T_1_AM acts as inverse agonist at hTAAR5

We observed that both hTAAR5 and mTaar5 show agonist-independent IP_3_ signaling, and this activity is not blocked by PTX treatment (Fig. 2C). Notably, for the first time, inverse agonism of 3-T_1_AM at hTAAR5 was demonstrated, since basal IP_3_ signaling of hTAAR5 is reduced after 3-T_1_AM application (Fig. 3B). Inverse agonism is a well-known mechanism of ligand action observed for constitutively active receptors [50] and has great therapeutic significance as a variety of clinically used drugs like β-blockers [51] and anti-allergic drugs targeting the histamine H1 receptor [52] act as inverse agonists [53,54]. The potential antidepressant S32212 showed inverse agonistic action at the serotonin receptor 2C in reducing basal IP_3_ production mediated via G_q/11_ activation [55].

### Differences in signaling properties of mTaar5 and hTAAR5

Our experiments on possible signaling pathways of TAAR5 after application of 3-T_1_AM revealed different signaling properties between mTaar5 and hTAAR5 since 3-T_1_AM shows inverse agonistic action at hTAAR5 in IP_3_ and MAP kinase activation but not at mTaar5 (Table 1, Fig. 3B, 3D). Additionally, we confirmed the volatile amine DMEA as an agonist for mTaar5 [8,20], but not for hTAAR5 (Fig. 2B), however we cannot rule out that other ligands like trimethylamine might activate Gs signalling [44]. A possible difference in ligand specificity between hTAAR5 and mTaar5 was already suggested by previous studies [44]. Additionally, DMEA might weakly bind to hTAAR5 in the 3-T_1_AM-induced signaling state as revealed by co-stimulation studies, while 3-T_1_AM has no modulatory effect on DMEA induced inverse agonistic action on mTaar5 (S1 Fig.).

The increased receptor activity in G_s_ pathway for mTaar5 might be explained by the fact that the mouse receptor is less constrained, which results in a certain pre-activation (basal signaling). The basally active conformation should have a higher affinity for the agonistic DMEA, which led to a simplified shift towards the activated receptor proportions (lowered energetic barrier), and this facilitates receptor activation by DMEA. hTAAR5 does not show a basal cAMP activity which likely impedes activation of G_s_ by this receptor compared to mTaar5.

However, both mouse and human TAAR5 show basal activities for G_q/11_ and MAP kinase signaling which points also to similarities in their pharmacological properties (Table 1). The main difference can be described by the basal activity for G_s_ signaling (only at mTaar5) and the higher MAP kinase signaling at hTAAR5 compared to mTaar5. Our studies on chimeric receptors revealed that details in the amino acid constitution (ECL2 and ICL3) and interactions at the extra- and intracellular sites likely contribute to the determination and fine-tuning of basal activities, probably by regulation of conformational differences.

## Conclusion

Our findings may imply that data obtained from mouse experiments are not simply transferable to humans, or vice versa, at least for TAAR5. Consequently, one should take into consideration that compounds tested in mouse models might have different, undesired, or even lack effects in humans. This is even more important considering that 3-T_1_AM is currently qualified as promising neuro- and cardio-protective compound against tissue injury after a stroke or a heart attack because of its extensive and rapid effects on body temperature in rodents [56,57]. Testing 3-T_1_AM-mediated effects on other TAAR subtypes or related GPCR will help to further decipher whether these effects are restricted to TAAR1 and hTAAR5. Whether 3-T_1_AM also couples to other receptors than TAARs may be analyzed by an independent series of systematic binding and signaling studies.

We present evidence for inverse agonistic action of hTAAR5 but not mTaar5 after 3-T_1_AM stimulation in our *in vitro* experiments. Based on these results, we propose that mTaar5 may not be involved in known 3-T_1_AM-induced pharmacological or physiological effects *in vivo*, since mTaar5 lacks any stimulating signaling properties after 3- T_1_AM application *in vitro*. However, one cannot rule out that mTaar5 might act differently *in vivo* compared to *in vitro* or that the observed pharmacological effects are mediated by other signaling pathways activated by locally elevated cAMP levels. It might be possible that, *in vivo*, TAAR5 forms hetero-oligomers with other receptors and thereby induces G-protein dependent signaling. Another possibility, for the *in vivo* situation, is that 3-T_1_AM has merely a modulatory effect on receptor signaling induced by other, so far not tested potential ligands of TAAR5. Thyronamines are thought to interact with the adrenergic system [58,59], as 3-T_1_AM also binds to the alpha2A adrenergic receptor [31]. It is also important to consider that the specificity for a respective G protein is influenced by several parameters such as i. agonist concentration, ii. expression level of the receptor [60,61], or iii. the cell type [62,63]. Further studies are necessary to reveal a more complete spectrum of 3-T_1_AM-induced signaling. TAARs are suggested to interfere with neurological disorders such as Parkinson´s disease, schizophrenia or Alzheimer´s disease [64]. Therefore, unraveling the full spectrum of evoked signaling effects of TAARs is a prerequisite for the development of specific and selective ligands as future treatment option.

## Supporting Information

S1 Fig DMEA is an inverse agonist on mTaar5 but not on hTAAR5. HEK293 cells transiently expressing hTAAR5 or mTaar5 were incubated without ligand or 10 μM 3-T_1_AM and/or 100 μM DMEA. Results are presented as relative light units (RLU). Data were obtained from 3 to 6 independent experiments measured in triplicates and are shown as mean ± SEM. An unpaired two-tailed Welsh-corrected t-test was used for statistical analyses; *p ≤ 0.05; **p ≤ 0.01; ***p ≤ 0.001.
(TIF)
